# Clinical Applicability of the Specific Risk Score of Dementia in Type 2 Diabetes in the Identification of Patients with Early Cognitive Impairment: Results of the MOPEAD Study in Spain

**DOI:** 10.3390/jcm9092726

**Published:** 2020-08-24

**Authors:** Angel Michael Ortiz Zuñiga, Rafael Simó, Octavio Rodriguez-Gómez, Cristina Hernández, Adrian Rodrigo, Laura Jamilis, Laura Campo, Montserrat Alegret, Merce Boada, Andreea Ciudin

**Affiliations:** 1 Institut de Recerca Vall d’Hebron, Universitat Autònoma de Barcelona (VHIR-UAB), 08035 Barcelona, Spain; a.ortiz@vhebron.net (A.M.O.Z.); cristina.hernandez@vhir.org (C.H.); 2 CIBER de Diabetes y Enfermedades Metabólicas Asociadas, Instituto de Salud Carlos III, 28029 Madrid, Spain; 3 Research Center and Memory Clinic, Fundació ACE, Institut Català de Neurociències Aplicades, Universitat Internacional de Catalunya, 08028 Barcelona, Spain; orodriguez@fundacioace.org (O.R.-G.); malegret@fundacioace.com (M.A.); 4 GMV Soluciones Globales Internet SAU, 28760 Valencia, Spain; arodrigo@gmv.com (A.R.); ljamilis@gmv.com (L.J.); 5 International Corporate Affairs, Alzheimer’s Disease, Eli Lilly and Co., 50019 Firenze, Italy; campo_laura@lilly.com; 6 Networking Research Center on Neurodegenerative Diseases (CIBERNED), Instituto de Salud Carlos III, 28029 Madrid, Spain

**Keywords:** type 2 diabetes, mild cognitive impairment, Alzheimer’s disease

## Abstract

Introduction: Although the Diabetes Specific Dementia Risk Score (DSDRS) was proposed for predicting risk of dementia at 10 years, its usefulness as a screening tool is unknown. For this purpose, the European consortium MOPEAD included the DSDRS within the specific strategy for screening of cognitive impairment in type 2 diabetes (T2D) patients attended in a third-level hospital. Material and Methods: T2D patients > 65 years, without known cognitive impairment, attended in a third-level hospital, were evaluated. As per MOPEAD protocol, patients with MMSE ≤ 27 or DSDRS ≥ 7 were referred to the memory clinic for complete neuropsychological assessment. Results: 112 T2D patients were recruited. A total of 82 fulfilled the criteria for referral to the memory unit (43 of them declined referral: 48.8% for associated comorbidities, 37.2% lack of interest, 13.95% lack of social support). At the Fundació ACE’s Memory Clinic, 34 cases (87.2%) of mild cognitive impairment (MCI) and 3 cases (7.7%) of dementia were diagnosed. The predictive value of DSDRS ≥ 7 as a screening tool of cognitive impairment was AUROC = 0.739, *p* 0.024, CI 95% (0.609–0.825). Conclusions: We found a high prevalence of unknown cognitive impairment in TD2 patients who attended a third-level hospital. The DSDRS was found to be a useful screening tool. The presence of associated comorbidities was the main factor of declining referral.

## 1. Introduction

The prevalence of dementia is rapidly increasing in developed countries due to social and demographic changes. This trend is expected to worsen in the coming decades, with the number of cases possibly even tripling in the next 25 years. In fact, the World Health Organization has declared dementia control a global health priority [1]. Although the effectiveness of treatment strategies is limited, there is increasing evidence that early diagnosis leads to significant economic and social benefits [2]. In addition, the disappointing results of clinical trials carried out in individuals with dementia have generated the hypothesis that interventions have occurred too late in the disease process. Probably the earlier we act, the better the chances of success [3]. Therefore, from the drug development point of view, an early diagnosis is critically needed to identify optimal candidates for new clinical trials. However, it is clear that in most health systems, dementia is underdiagnosed, and when diagnosis occurs, it is typically at a relatively late stage in the disease process. Mild cognitive impairment (MCI) represents a greater decline in cognition to that observed in normal cognitive decline associated with age but not severe enough to cause significantly impaired daily function [4,5]. Generally, amnestic MCI is characterized by a decreased ability to learn new information or retrieve stored information, without affecting the basic activities of the daily living. Despite the fact that patients with MCI can conduct a normal life, the important problem associated with MCI is that about 10–30% will progress annually to dementia [6]. At present, there are no reliable indicators that could help us identify which patients are at higher risk of progression to dementia.

Current epidemiological data show that patients with T2D have 2–3 times more risk of developing Alzheimer’s disease (AD) than the non-diabetic population matched by age and others established risk factors for developing dementia [5,6]. Furthermore, the diabetes duration, the poor glycemic control and the frequency and severity of hypoglycemia are independent risk factors associated with an accelerated cognitive decline in people with T2D [7,8,9,10,11].

Early diagnosis of MCI is important in T2D population for several reasons. First, the early detection may be able to establish interventions aimed at slowing or even preventing progression to dementia. In this regard, the use of drugs with no or only minimal hypoglycemia capacity is strongly recommended [9]. In fact, the American Diabetes Association ADA clinical guidelines recommend screening for early detection of cognitive impairment in adults older than 65 at the initial visit and annual follow-ups as appropriate [10]. In addition, early diagnosis can allow the patient to participate in research studies and support groups in the community if desired [11]

In recent years, several dementia prediction scores have been developed in the diabetic population, such as the *Diabetes Specific Dementia Risk Score* (DSDRS) [12]. The variables collected are clinical and do not require the patient’s presence. These variables are age, the presence of micro/macrovascular disease, acute events related to diabetes, depression and the level of formal education of the patient. After the score calculation, a result with predictive value of developing dementia in 10 years is obtained. The applicability has not been widely extended.

On this basis, the objectives of the present study were (1) to evaluate the results of pre-screening for MCI in the T2D population within the MOPEAD project in Spain and (2) to evaluate the applicability of DSDRS as a screening tool for T2D patients with cognitive impairment.

## 2. Materials and Methods

This study is part of the MOPEAD project: Models of Patient Engagement for Alzheimer’s Disease. It is a European Innovative Medicines Initiative (IMI2) Project-grant number 115985. The project evaluated four models of patient engagement strategy (Runs) aimed to help identify individuals at risk of AD in a multicenter setting (five countries were analyzed). The specific objectives were to (a) identify individuals in the community with hidden cognitive impairment or individuals without cognitive impairment at risk of AD; (b) promote cognitive wellbeing and healthy cognitive aging in the European Union using AD Citizen Science, Open House and developing campaigns in primary and tertiary care setting; (c) test innovative patient engagement models and generate clinical and demographic data to evaluate the most efficient approaches; (d) develop common pre-screening methodologies for practitioners.

In each country, at least 100 patients were to be screened/model in order to refer at least 33 patients to the memory clinic/model. The four different models were called “Runs”, as follows: (a) Run 1: AD Citizen Science, on-line screening campaign; (b) Run 2: Open House campaign; (c) Run 3: Primary care based patient engagement; (d) Run 4: Tertiary care based patient engagement. The extended protocol was previously published and explained [13].

This paper focuses on a prospective observational study performed only in one center (Vall d’Hebron University Hospital (HVH), Barcelona, Spain) as the leader of Run-4 (Tertiary care–based patient engagement: Diabetologist setting) of the MOPEAD study. The study was approved by the local ethics committee and conducted following the Strengthening the Reporting of Observational Studies in Epidemiology guidelines. A total of 112 consecutive T2D patients fulfilling the inclusion criteria were recruited from the pool of patients that attended the outpatient clinic. As per MOPEAD protocol, the inclusion criteria were (a) age between 65–85 years; (b) T2D with duration > 5 years; (c) functionally literate, without severe auditory and visual abnormalities; (d) written informed consent; (e) clinical data available for the DSDRS calculation. Exclusion criteria were (a) other types of diabetes; (b) previous diagnosis of AD, MCI or other types of dementia following the diagnostic criteria of the NIA-AA [14]; (c) history of stroke; (d) history of unstable neurological or psychiatric conditions that may affect cognition, including the presence of depression; (e) severe metabolic or systemic disease such as unstable acute cardiovascular disease, renal failure with GFR < 30 mL/min/m^2,^ decompensated liver cirrhosis or liver failure, hypothyroidism untreated or vitamin B12 deficiency; (f) drugs affecting cognitive status, for example, antipsychotics, opioids, long half-life benzodiazepines like diazepam; (g) uncorrected severe sensory deficits (blindness, deafness).

All participants underwent a complete medical history, physical examination and biochemical analysis.

DSDRS was calculated based on the clinical data and the medical history, as described by Exalto et al. [12]. The score is the sum of several points that are assigned as follows: (a) age (60–64 years: 0, 65–69 years: 3, 70–74 years: 5; 75–79 years: 7; 80–84 years: 9; >85 years: 10), (b) associated conditions (acute metabolic event: 2; macrovascular disease: 1; diabetic foot: 1; cerebrovascular disease: 2; cardiovascular disease: 1; depression: 2), (c) education level (<12 years: 0; >12 years: −1). The acute metabolic event was defined as severe hypoglycemia requiring assistance from another person, hiperosmolar hyperglycemia or ketoacidosis. The complete score calculation is available in Supplementary Material, Annex 2.

All the patients underwent the Minimental State Evaluation questionnaire (MMSE) [15], which was administered by the same member of the health-care team for all patients. During the visit, all the patients were asked up to three initial questions about their cognitive state: (1) “Do you feel that your memory is getting worse?” (2) “How long have you been noticing it?” and (3) “Are you worried about this alteration?” Additionally, a hypoglycemia survey was created for the MOPEAD protocol in order to evaluate the presence, the number and severity of hypoglycemia (see Supplementary Material). As per MOPEAD protocol, [13] those subjects having a MMSE score ≤ 27 or DSDRS ≥ 7 and positive answer for ≥ 2 of the initial questions or DSDRS ≥ 10 were referred to the specialized memory clinic (Fundació ACE, Barcelona, Spain) to complete the neuropsychological evaluation.

**Statistical analysis:** The categorical variables were expressed as a percentage. For the quantitative variables, means and standard deviation are displayed if they follow a normal distribution, and those that do not are displayed in median and range. To evaluate differences between groups Chi square test was used for qualitative variables and Analysis of Variance (ANOVA); following this, DMS post-hoc tests for quantitative variables were used. For variables that do not follow a normal distribution, a nonparametric test was used to compare between groups (Kruskal–Wallis). To evaluate the correlation between MMSE and DSDRS, the Pearson correlation test was performed. Significance was accepted at *p* < 0.05. Regression was used to predict the Receiver Operating Characteristic (ROC) curves and the chi-square test for comparison of ROC area. The statistical analyses were performed with the statistical package SPSS version 21. Data of all the patients included in the study was used for descriptive statistics. Data from the patients that underwent a complete neuropsychological evaluation at the memory clinic was used for correlations and regression analysis.

## 3. Results

A total of 112 consecutive T2D patients were recruited. The baseline characteristics are presented in Table 1. Of all the enrolled patients, 82 (73.12%) fulfilled criteria for referral to the Fundació ACE’s memory clinic for suspected cognitive impairment as per MOPEAD protocol.

Patients who met criteria for referring to memory clinic were older (median of 75 years (65–86) versus 68 years (65–79), *p* = 0.02) and had a higher percentage of insulin treatment (73.1% versus 60%, *p* = 0.04) than patients who did not. In addition, a trend to present an increased burden of cardiovascular disease and longer diabetes duration was also observed. Furthermore, as expected, this group of patients presented lower MMSE (26.84 ± 2.01 versus 28.8 ± 0.66, *p* = 0.04) and higher DSDRS scores (7.48 ± 2.2 versus 4.63 ± 1.2, *p* = 0.02), respectively. The detailed number of patients that fulfilled the criteria for referral to the memory clinic (MMSE or DSDRS or both) are presented in Supplementary Material, Annex 1. All the patients had at least two positive answers to the initial questions.

**Figure 1:**
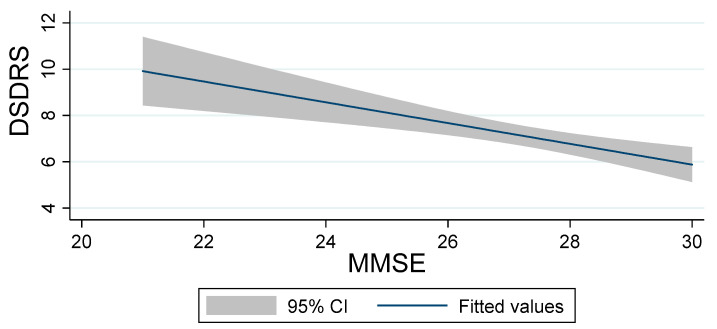
Correlation between DSDRS and MMSE scores. DSDRS: diabetes specific dementia risk score, MMSE: Mini-Mental State Examination.

Pearson correlation analyses showed inversely proportional relationship between MMSE and DSDRS scores (r = −0.3640; CI (−0.414 – −0.135) *p* < 0.05), as reflected in Figure 1.

Of the 82 T2D patients meeting criteria of referral to memory clinic, 43 (52.4%) declined participation in the second phase of the study due to associated comorbidities (21, 48.8%), lack of interest (16, 37.2%), and absence of social support (6, 13.95%).

A total of 39 T2D were evaluated at the memory clinic and underwent a complete neuropsychological assessment, as previously described [13], and 34 individuals (87.2%) received a diagnosis of MCI, 3 (7.7%) of AD dementia and 2 (5.1%) of cognitively healthy. Table 2 reflects the characteristics of these patients.

The regression logistic analysis revealed that MMSE and DSDRS scores were independent predictors of the global cognitive impairment (MCI plus dementia), as reflected in Table 3.

**Figure 2:**
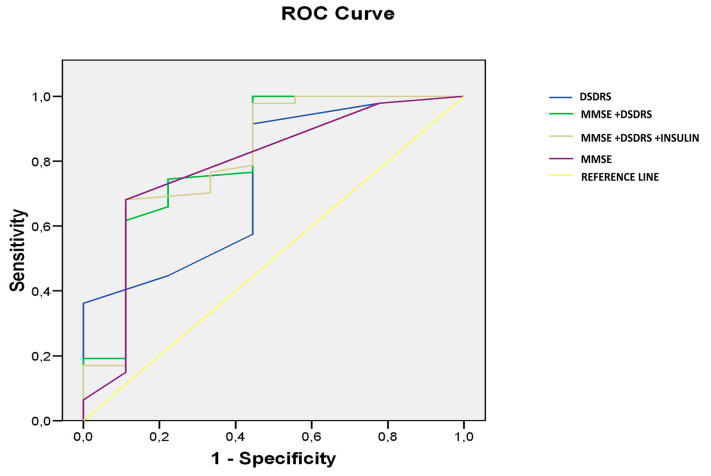
The ROC Curve for the MMSE and DSDRS as screening tools of cognitive impairment. MMSE: Mini-Mental State Examination, DSDRS: diabetes specific dementia risk score.

The predictive value of DSDRS ≥ 7 for the diagnosis of cognitive impairment was significant AUC: 0.739, CI 95% (0.557–0.921), *p* < 0.024 (Figure 2).

The prediction capacity of combined MMSE and DSDRS for identifying subject with cognitive impairment (MCI + dementia) was AUROC 0.902 (*p* 0.003, CI 95% (0.840–0.992)), significantly higher (*p* 0.01) than MMSE (AUROC 0.785, *p* 0.007, CI 95% (0.814–0.948)) or DSDRS separately (AUROC 0.739, *p* 0.024, CI 95% (0.609–0.825)) (Figure 2). No significant differences were seen between MMSE and DSDRS separately. When insulin use was added to the model, the predictive value of the scores combined was not changed (AUROC 0.904, *p* 0.001, CI95% (0.841–0.966)).

## 4. Discussion

In the present study, we found a high prevalence of cognitive impairment in the T2D patients older than 65 who attended a third-level hospital, as part of the MOPEAD protocol in Spain. The suspected diagnostic was confirmed in a reference memory clinic, and the clinical DSDRS score showed a significant predictive value of cognitive impairment.

Although the DSDRS was not designed as a screening tool, our results suggest that it might eventually be used as a complementary test for this purpose. Most of the developed countries are using electronic medical history, thus making largely available reliable data in the medical records regarding age, level of formal education, history of acute metabolic events, history of diabetic foot, history of microvascular disease, history of cerebrovascular disease, history of cardiovascular disease, diagnosis of depression. This information permits us to calculate DSDRS easily. An interesting proposal could be the automatic calculation of the DSDRS score during or even before the patient’s visit. In this regard, it could be extremely useful in clinical practice to receive a warning alarm in the event that the diabetic patients present a score ≥ 7 in order to facilitate the identification of patients at higher risk of presenting cognitive impairment. This strategy would allow selecting those patients in whom a specific assessment of cognitive status should be implemented. However, specific studies to confirm these preliminary results in larger cohorts are needed.

Unrecognized cognitive dysfunction can affect treatment adherence and diabetes self-management resulting in poor glycemic control, an increased frequency of severe hypoglycemic episodes, and hospital admissions [16]. Regarding hypoglycemia, two independent studies [17,18] showed that three or more severe hypoglycemic episodes increased by almost two-fold the risk of dementia in T2D patients. Despite of the role of hypoglycemia in the cognitive function, the frequency and severity of hypoglycemia does not appear in current questionnaires for the evaluation of the risk of developing dementia, and therefore, this crucial point should be tackled urgently. In order to fill this gap, we have designed a hypoglycemia survey (see Supplementary Material) which was administered to all the patients included in the present study. Notably, 6 patients who fulfilled the criteria of cognitive impairment presented a history of severe hypoglycemia while no cognitively healthy participant presented hypoglycemia.

In our study, the prevalence of MCI was 87.2% and for AD 7.7% in the group of patients that attended the memory clinic. The prevalence of MCI in our sample was calculated based on the 39 patients that attended the memory clinic. However, it should be noted that more than half of the patients that met criteria for referral to the memory clinic declined to continue the study, mainly due to associated comorbidities and lack of interest. At present, the real prevalence of MCI in T2D patients is unclear. Gao Y et al. reported a prevalence of MCI in T2D of 62.2% and AD 11.9% in Chinese population [19] while Albai et al. [20] reported 42.03% MCI in T2D patients having a mean age of 63 years (range 57–71 y). The mean age of patients included in our study was of 75 years, all of them were patients recruited from a tertiary care setting and most of them presented co-morbidities. These factors should be taken into account when comparing the different series reported in the literature. It should be noted that the neuropsychological battery and the data reported was not homogeneous between the studies. Gao et al. and Albai et al. did not report data on the MMSE score or details on the neuropsychological battery that was used. Additionally, Albai et al. did not report data on the education level (one of the most important variables that influences cognitive function scoring), and both cited studies lack data on the cardiovascular risk factors, complications of T2D and hypoglycemia.

Furthermore, the population included in our study was selected from the patients attended in a tertiary setting, showing a high prevalence of MCI among this population, while the other two studies included patients from general population. At present, there is no data regarding the prevalence of cognitive impairment in T2D patients attended in a third level hospital, which represents a strength of our study, even if we have preliminary results that need to be validated.

Patients attended in a third-level hospital usually are plurimedicated. They usually are prescribed complex treatment regimes, some of them including insulin. As reflected by Table 3, almost 70% of the patients were using insulin. The importance of early detection of cognitive impairment in this population comes from the need to adapt and adjust the specific treatment for T2D to the capacity of management of the patient, in order to avoid worsening of T2D associated complications and to limit possible errors in the administration of the medication, which may lead to hypoglycemia or other unfavorable and potentially life-threatening events.

Several neuropsychological questionnaires have been proposed for the screening of cognitive decline in T2D population [21]. However, the number of patients whose cognitive function needs evaluating by the general practitioner or the endocrinologist/diabetologist is potentially enormous, and a simpler and more cost-effective case-finding strategy to detect undiagnosed cognitive impairment is needed. In our study, we showed that a simple score (DSDRS) calculated based on clinical variables is useful as a screening tool for cognitive impairment (AUROC 0.739, *p* 0.024, CI 95% (0.609–0.825)) [12]. No differences were seen between the predictive values of MMSE and DSDRS separately as screening tools for cognitive impairment. Nevertheless, as mentioned, the novelty of the DSDRS as a screening tool is that it can be calculated using existing data in the medical history of the patient. Patients with DSDRS ≥ 7 can be candidates for a more specific study of cognitive function. This score consists of several clinical and demographic variables (age, gender, education, history of diabetic foot, acute metabolic events, depression, microvascular disease, cardiovascular disease and cerebrovascular disease). It should be noted that in our study, as per MOPEAD protocol, patients with stroke or depression were excluded. We admit that in the real world, the DSDRS could be higher than the obtained in the present study. Nevertheless, even with this bias, our results showed that the DSDRS was a useful tool for identifying patients with diabetes at risk of dementia. The DSDRS was designed as a risk score of dementia at 10 years, ranging from a 5% of dementia risk for those with the lowest score up to a 73% risk for the highest score. The DSDRS was not designed as a screening tool, but it might eventually be used as a complementary test for this purpose, in particular, if more detailed information regarding diabetic complications (i.e., degree of diabetic retinopathy) and glycemic control (i.e., hypoglycemic events and glycemic variability) was added. When the DSDRS score was added to the MMSE, the predictive value for cognitive impairment significantly improved, supporting the hypothesis that DSDRS can be an useful complementary screening tool (AUROC 0.902 (*p* 0.003, CI 95% (0.840–0.992)), significantly higher than MMSE (AUROC 0.785, *p* 0.007, CI 95% (0.814–0.9648)) or DSDRS separately (AUROC 0.739, *p* 0.024, CI 95% (0.609–0.825)).

One limitation of our study is the small sample size (as per protocol 100 patients/country [13]) and the fact that most of the patients that met criteria for referral to the memory clinic declined further participation in the study due to associated comorbidities and lack of interest. This observation deserves a comment: The study of cognitive status is not a current priority for the patients and health care providers. This is a significant gap that should urgently be filled due to the importance of the early detection of cognitive impairment and the implications in the management of the complex treatment regimes.

Our results are preliminary, and a specific study to confirm that a refined DSDRS score could be a useful complementary test for identifying T2D patients who should be referred to a memory clinic is needed. If our results are confirmed in a further study, the DSDRS could be a screening tool that might easily be implemented and automatically calculated by the electronic medical records of the patients, as part of the daily clinical practice.

## 5. Conclusions

Diabetic patients in the tertiary care setting seem to have a high risk of developing cognitive impairment, but they are usually patients with other severe diseases and complications. In consequence in the case of these patients, the cognitive impairment is viewed as a secondary issue. Nevertheless, early detection of AD is particularly important in this scenario because it could have a great impact on diabetes control and self-management of complex regimes of treatment. Therefore, reliable screening tools and more education about cognitive impairment as a complication of type 2 diabetes are needed both for patients and diabetes care providers.

## Figures and Tables

**Table 1:** Baseline characteristics of the patients included in the study.

	Meeting Criteria for Referral to Memory Clinic	Not Meeting Criteria for Referral to Memory Clinic	*p*
*N* (%)	82 (73.2%)	30 (26.7%)	0.001
Age (years) mean ± SD	74.77 ± 4.54	69.47 ± 3.44	0.02
Sex (women) *n (*%)	36 (43.9%)	13 (43.3%)	0.72
Education level (years) mean ± SD	6.87 ± 3.25	6.8 ± 4.73	0.46
BMI (kg/m^2^) mean ± SD	28.9 ± 5.1	32.0 ± 5.8	0.09
Smoker *n* (%)	72 (87.8%)	29 (96.6%)	0.06
Hypertension *n* (%)	77 (93.9%)	27 (90%)	0.63
Dyslipidemia *n* (%)	76 (92.6%)	29 (96.6%)	0.21
Obstructive sleep apnea *n* (%)	17 (20.7%)	8 (26.6%)	0.35
Ischemic heart disease *n* (%)	25 (30.4%)	5 (16.1%)	0.06
Peripheral arteriopathy *n *(%)	20 (24.3%)	4 (13.3%)	0.08
T2D duration (mean ± SD)	19.7 ± 9.8	17.5 ± 7.8	0.09
Insulin use *n* (%)	60 (73.1%)	18 (60%)	0.04
HbA1C (%) mean ± SD	7.7 ± 1.0	7.4 ± 0.8	0.43
Severe hypoglycemia (*n*)	6	0	NA
Diabetic retinopathy *n* (%)	53 (64.3%)	15 (50%)	0.28
Diabetic nephropathy *n* (%)	34 (41.4%)	13 (43.3%)	0.64
Diabetic polyneuropathy *n* (%)	27 (32.9%)	9 (30%)	0.53
MMSE mean ± SD	26.84 ± 2.01	28.8 ± 0.66	0.04
DSDRS mean ± SD	7.48 ± 2.2	4.63 ± 1.2	0.02

BMI: body mass index, T2D: type 2 diabetes, HbA1C: glycated hemoglobin A1C, MMSE: mini mental state evaluation, DSDRS: diabetes specific dementia risk score.

**Table 2:** Characteristics of the patients that were performed a complete neuropsychological evaluation at the memory clinic.

	Patients That Were Evaluated at the Memory Clinic
*N*	39
Race	Caucasian
Age (years) median (min–max)	75 (68–84)
Education level < 9 years %	83.87
Sex (women) %	44.1
BMI (kg/m^2^) mean ± SD	28.51 ± 4.01
Smoker (%)	54.83
Hypertension (%)	87.09
Dyslipidemia (%)	96
Ischemic heart disease (%)	25.80
Peripheral arteriopathy (%)	19.2
T2D duration (mean ± SD) years	19.1 ± 6.2
Insulin use%	67.74
HbA1C (%) mean ± SD	7.69 ± 0.76
Hypoglycemia (*n*)	64
Severe hypoglycemia (*n*)	6
Diabetic retinopathy (%)	54.83
Diabetic nephropathy (%)	38.70
Diabetic polyneuropathy (%)	22.58
MMSE (mean ± SD)	26.72 ± 2.14
Prevalence of MCI%	87.2

BMI: body mass index, MCI: mild cognitive impairment, MMSE: Mini-Mental State Examination, DSDRS: diabetes specific dementia risk score.

**Table 3:** Independent predictors of cognitive function (MCI and dementia).

Coefficients ^a^						
Model		Unstardardized Coefficients		Standardized Coefficients	t	Sig.
		B	Error Estándar	Beta		
1	(Constant)	6.147	1.205		5.102	0.000
	Age	−0.012	0.013	−0.121	−0.895	0.373
	Insulin use	0.098	0.093	0.088	1.054	0.295
	MMSE score	−0.149	0.025	−0.528	−6.021	0.000
	DSDRS score	0.063	0.027	0.306	2.310	0.023

^a^ Dependent variable: Cognitive impairment. MMSE: Mini-Mental State Examination, DSDRS: diabetes specific dementia risk score.

## Supplementary Materials

The following are available online at https://www.mdpi.com/2077-0383/9/9/2726/s1. Hypoglycemia survey, 2. Annex 1, 3. Annex 2.
