# Supplementary material for: Clinical Applicability of the Specific Risk Score of Dementia in Type 2 Diabetes in the Identification of Patients with Early Cognitive Impairment: Results of the MOPEAD Study in Spain
Source: J Clin Med. 2020 Aug 24;9(9):2726. doi: 10.3390/jcm9092726 (PMC7565958; doi:10.3390/jcm9092726)
Supplement: Supplementary file 1 [file jcm-09-02726-s001.pdf]

Table S1. Patients referred to the Memory Clinic

Criteria for referral to Memory Clinic, as per MOPEAD Protocol (as explained in the text): MMSE score ≤ 27 or DSDRS≥ 7 and positive answer for ≥ 2 of the initial questions or DSDRS ≥ 10

	YES (N=82)	NO (N=30)
MMSE≤27 + DSDRS<7	16	0
MMSE>27+DSDRS≥7	32	0
MMSE≤27+DSDRS≥7	34	0
MMSE>27+DSDRS<7	0	30
Total	82	30

Table S2. Confirmed cognitive impairment at the memory clinic: 39 patients attended of the 82 that were referred

	Cognitive impairment (37)	Normocognitive (2)
MMSE≤27 + DSDRS<7	4	1
MMSE>27+DSDRS≥7	14	1
MMSE≤27+DSDRS≥7	19	0
MMSE>27+DSDRS<7	0	0
Total	34	2

Supplementary material-Annex 2. Diabetes Specific Dementia Risk Score calculation

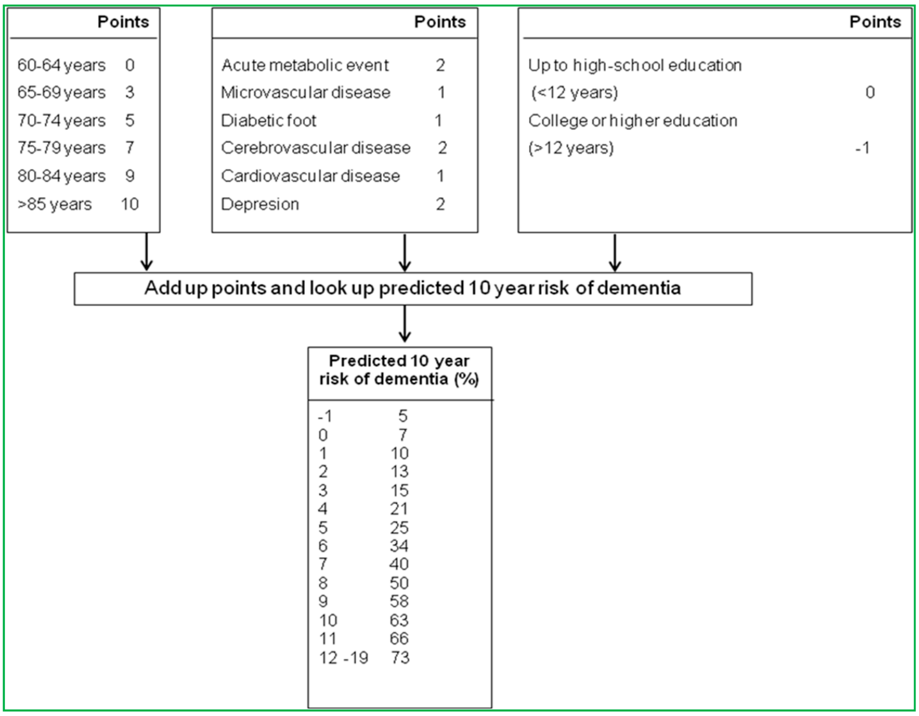

Reference: L. G. Exalto *et al.*, "Risk score for prediction of 10 year dementia risk in individuals with type 2 diabetes: a cohort study," *Lancet. Diabetes Endocrinol.*, vol. 1, no. 3, pp. 183–190, Nov. 2013, doi: 10.1016/S2213-8587(13)70048-2.
